# Mitochondrial and oxidative stress genes are differentially expressed in neutrophils of sJIA patients treated with tocilizumab: a pilot microarray study

**DOI:** 10.1186/s12969-016-0067-7

**Published:** 2016-02-09

**Authors:** Ebun Omoyinmi, Raja Hamaoui, Annette Bryant, Mike Chao Jiang, Trin Athigapanich, Despina Eleftheriou, Mike Hubank, Paul Brogan, Patricia Woo

**Affiliations:** Infection, Inflammation and Rheumatology Section, Institute of Child Health, UCL, London, UK; Centre for Adolescent Rheumatology, University College London, 4th Floor, Rayne Building, 5, University Street, London, WC1E 6JF UK; UCL Genomics, Institute of Child Health, London, UK

**Keywords:** Systemic juvenile idiopathic arthritis (sJIA), Anakinra, Tocilizumab, Gene expression, Neutrophils

## Abstract

**Background:**

Various pathways involved in the pathogenesis of sJIA have been identified through gene expression profiling in peripheral blood mononuclear cells (PBMC), but not in neutrophils. Since neutrophils are important in tissue damage during inflammation, and are elevated as part of the acute phase response, we hypothesised that neutrophil pathways could also be important in the pathogenesis of sJIA. We therefore studied the gene profile in both PBMC and neutrophils of sJIA patients treated with tocilizumab.

**Methods:**

We studied the transcriptomes of peripheral blood mononuclear cells (PBMC) and neutrophils from eight paired samples obtained from 4 sJIA patients taken before and after treatment, selected on the basis that they achieved ACR90 responses within 12 weeks of therapy initiation with tocilizumab. RNA was extracted and gene expression profiling was performed using Affymetrix GeneChip Human Genome U133 Plus 2.0 microarray platform. A longitudinal analysis using paired t-test (*p* < 0.05 and FC ≥ 1.5) was applied to identify differentially expressed genes (DEGs) between the two time points followed by ingenuity pathway analysis. Gene Set Enrichment Analysis (GSEA) and quantitative real-time PCR were then performed to verify the microarray results.

**Results:**

Gene ontology analysis in neutrophils revealed that response to tocilizumab significantly altered genes regulating mitochondrial dysfunction and oxidative stress (*p* = 4.6E-05). This was independently verified with GSEA, by identifying a set of oxidative genes whose expression correlated with response to tocilizumab. In PBMC, treatment of sJIA with tocilizumab appeared to affect genes in Oncostatin M signalling and B cell pathways.

**Conclusions:**

For the first time we demonstrate that neutrophils from sJIA patients responding to tocilizumab showed significantly different changes in gene expression. These data could highlight the importance of mitochondrial genes that modulate oxidative stress in the pathogenesis of sJIA.

**Electronic supplementary material:**

The online version of this article (doi:10.1186/s12969-016-0067-7) contains supplementary material, which is available to authorized users.

## Background

Juvenile Idiopathic Arthritis (JIA) is a heterogeneous group of arthritides classified into 7 subtypes by the International League of Associations for Rheumatology (ILAR) [1]. We have previously examined the transcriptome of patients with systemic JIA (sJIA), a distinct clinical subgroup with characteristic systemic inflammation as well as arthritis [2]. Systemic inflammatory features of this group of patients include quotidian fever, evanescent skin rash, lymphadenopathy, serositis and hepatosplenomegaly. These clinical features are reminiscent of the heritable autoinflammatory diseases; consequently, sJIA is frequently referred to as an autoinflammatory disease because of these clinical manifestations, lack of autoantibodies, and association with variants in inflammatory and anti-inflammatory cytokine genes, including IL-1, IL-6, and IL-10 [3–8]. High or abnormal production of inflammatory cytokines in the plasma, and impressive results from clinical trials to modify IL-1 and IL-6 cell signalling have provided further evidence for the important roles of these cytokines in the pathogenesis of sJIA [9–17].

The molecular pathways leading to the symptoms of sJIA are not yet fully understood, but have been clarified to some extent during recent years by the use of microarray technology. So far, gene expression studies have been performed mostly on peripheral blood mononuclear cells (PBMC) from sJIA patients, with the exception of one study which examined whole blood [18]. Studies of this nature have led to the identification of gene clusters associated with disease activity [2] and severity [19], and revealed dysregulation of cytokine pathways [9, 12, 18]. A study applied DNA microarray methods across different subtypes of JIA, and identified patterns of gene expression that correlated with clinical characteristics of the different subtypes. In particular, it was shown that sJIA is distinct from Oligoarticular and Polyarticular JIA [20]. Furthermore, gene expression profiles in neutrophils from children with polyarticular JIA demonstrate defects in genes modulated by IFN-gamma and IL-8 [21–23].

Since many autoinflammatory diseases involve the genes and pathways of the innate immune system, we proposed that neutrophils were worthy of further study in sJIA. Neutrophils are highly specialised leucocytes that mediate inflammatory changes in tissues, and are important effectors of innate/early immune responses [24]. Once activated, neutrophils can produce reactive oxygen species that neutralise the effects of bacteria and fungi, and together with chemokines and other inflammatory mediators, orchestrate inflammation in tissues [24]. In autoinflammatory diseases, abundant neutrophils are usually seen in sites of inflammation, often in the relative absence of lymphocytes [25, 26]. Consequently, we hypothesised that neutrophils are important in the pathogenesis of sJIA, and that there would be important differences in the differentially expressed genes in response to tocilizumab.

## Methods

### Patients and treatment

This pilot study included symptomatic sJIA patients with disease duration ranging from 9 months to 7 years (Table 1), who were selected for analysis on the basis that they achieved an adapted American College of Rheumatology response of 90 (ACR90) to tocilizumab. The clinical features of the patients in this study are shown in Table 1. All patients provided written informed consent to enter this observational study, with ethical approval by Great Ormond Street Hospital for Children NHS Trust (GOSH)/Institute of Child Health Research Ethics committee (registration number 02RU06). We examined gene expression profiles before, and 3 months after treatment with tocilizumab (a humanised monoclonal antibody targeting the interleukin 6 receptor, received as routine clinical care) at GOSH. The patients received tocilizumab using a standard recommended dosing regimen [14, 16]; samples from responders were collected from November 2008 to July 2009. All patients fulfilled sJIA ILAR classification criteria [27]. Patients were considered for this study group if they had persistent signs of inflammation refractory to therapy with methotrexate (MTX), with or without corticosteroid or anti-TNF-α therapy. Blood samples referred to as “Before” were obtained from all patients at routine baseline screening prior to the initiation of treatment when the disease status was active. Twelve weeks from enrolment into the study, an “After” sample was collected. Clinical assessment of disease activity was determined using the JIA core set criteria and definition of improvement [28]. The American College of Rheumatology (ACR) paediatric adapted improvement score was then calculated at the time of sample collection (28). Response was defined as: paediatric-adapted American College of Rheumatology (ACR) 90 response, plus normalisation of the C reactive protein (CRP) and erythrocyte sedimentation rate (ESR).

**Table 1:** Clinical features of patients and response to tocilizumab

Sample code	37-5	37-7	50-2	50-4	51-1	51-3	53-2	53-4
Sample type	N	N	P, N	P, N	P, N	P, N	P, N	P, N
Conditions	Pre-TOC	12 weeks TOC	Pre-TOC	12 weeks TOC	Pre-TOC	12 weeks TOC	Pre-TOC	12 weeks TOC
Ethnicity	Caucasian		Caucasian		Asian		Caucasian	
Gender	Male		Female		Male		Female	
Age at onset (AAO)	7 years		9 years		14 years		4 years	
Disease duration	3.4 years		7 years		10 months		9 months	
Fever	Yes	No	No	No	No	No	Yes	No
Rash	Yes	No	No	No	No	No	No	No
Total WCC (lymphocytes) x 10^9^/L	22.22 (1.36)	10.76 (4.99)	10.51 (1.28)	5.58 (2.67)	N/D	5.84 (2.52)	13.98 (4.80)	8.09 (4.03)
CRP (mg/l), 0-20 normal range	84	<5	24	<5	13.6	<3	56	<5
ESR (mm/h), 0–10 normal range	65	<1	29	3	N/D	1	35	<1
Joint active	6	0	22	10	6	0	51	0
Joint limited motion	6	0	30	12	7	0	49	3
Parental VAS	N/D	0	6.8	1	2.8	0.4	5	2.2
Physician VAS	5	0	8	4	3	1	8	0.4
CHAQ	1.75	0	1.5	0.38	0	0	1.13	0.5
ACR	-	90	-	90	-	90	-	90

The demographics, clinical and laboratory parameters of the patients whose peripheral blood was used in this study are shown. For each patient, baseline measurements were taken before starting tocilizumab (TOC) treatment, and 12 weeks later. Normal range for total white cell count (WCC) is 4.5–13.5 × 10^9^/L and for lymphocytes 1.5–7 × 10^9^/L. The different types of samples taken/analysed were: PBMC = P; and Neutrophils = N. There are 3 paired samples for PBMC (P) and 4 for neutrophils (N). Response to treatment was determined by ACR90 response definition of improvement in juvenile arthritis [47], plus normalisation of the erythrocyte sedimentation rate (ESR) and C reactive protein (CRP). *N/D* not determined, *VAS* visual analogue score, *CHAQ* child health assessment questionnaire, *ACR* American College of Rheumatology

### Leucocyte separation

PBMC were obtained from Ficoll separation of whole blood using Lymphoprep reagent (Stemcell Technologies). Neutrophils were subsequently isolated from the granulocyte and red cell pellet formed at the bottom of the Ficoll tube by hypotonic cell lysis in ammonium chloride buffer. Cell viability was assessed by trypan blue dye exclusion. Purified cells were visualised and counted using a haemocytometer before re-suspension in TRIzol reagent (Invitrogen, Paisley, UK). Care was taken to minimize the time between blood-drawing and placing cells in TRIzol reagent to within 3 h.

### Assessment of purity and the effect of isolation process on neutrophil activation status

We used two-colour flow cytometric analysis to monitor ex vivo activation of neutrophils as a result of sample manipulation. Antibody staining for surface markers was performed as previously described [29], using PE conjugated mouse Anti-Human CD11b/Mac-1 (a marker to monitor ex vivo activation of neutrophils), and APC-Cy7 mouse Anti-Human CD16, expressed on the surface of neutrophils. A FACScan flow cytometer and CellQuest analysis software were used for the acquisition and analysis of the data. The purity and activation status of isolated neutrophils were evaluated by gating on the CD11b/Mac-1 and CD16 double-positive cells.

### Lipopolysaccharide-induced activation of isolated neutrophils

Isolated neutrophils at a density of 2 × 10^6^ cells/ml in RPMI were cultured at 37°C for 1 h in the presence or absence of 1μg/ml lipopolysaccharide (LPS). The level of neutrophil activation was again evaluated by flow cytometry analysis of CD11b/Mac-1 on CD16 positive cells.

### Microarray procedures

The protocols for RNA extraction and microarray hybridization to Affymetrix U133 plus 2.0 arrays (Affymetrix, Santa Clara, CA), which includes approximately 54,000 probe sets representing 47,400 human transcripts were as previously described [2]. Processing of Affymetrix data was performed in GeneSpring GX11.0 (Agilent) using GCRMA (RMA; Robust Multi-array Analysis that accounts for the probes GC content) method for normalizing and summarizing probe-level intensity measurements [30]. Probe sets with very low absolute expression intensity values (<10) in all patients in either the “before” or “after” samples were filtered out since at this level it would be difficult to distinguish a true effect from background noise [31]. Multiple probe sets mapping to the same gene that passed this filter were retained and the differential expression of any probe set for a given gene was used as a surrogate for differential gene expression. The affymetrix data files have been submitted to NCBI Gene Expression Omnibus (GEO; http://www.ncbi.nlm.nih.gov/geo/) under accession number GSE76492.

### Statistical analysis of microarray data

Paired t-test of the "before" and "after" treatment samples at statistical threshold of *p* < 0.05 with fold-change (FC) ≥1.5 was considered as significant for identification of differentially expressed genes. Both unsupervised and supervised hierarchical clustering was performed using Pearson’s centered correlation and Ward’s linkage rule within GeneSpring software to generate heatmaps of gene expression profiles.

### Ingenuity Pathway Analysis (IPA)

The differentially expressed probe sets identified with statistical testing were uploaded into the IPA knowledge database (version 7.2, Ingenuity® Systems, www.ingenuity.com) to explore the pathways that were significantly associated with each dataset. In the case of multiple probe sets mapping to the same transcript, IPA considered the probe set with the highest fold change for the pathway analysis. Each annotated gene was mapped to its corresponding gene object in the IPA Knowledge Base. The Affymetrix U133 plus 2.0 array was selected as background or reference dataset for calculation of significant functions/pathways. The core analysis was run using the following setting in IPA: data source included all species, tissues and cell lines; interactions were queried on all genes stored within IPA; and no fold-change cut-off was specified. The significance of the association between the data set and the canonical pathways was calculated in 2 ways according to IPA user’s manual: 1) a ratio of the number of genes from our dataset that map to the pathway, divided by the total number of genes that make up the pathway; 2) Fisher’s exact test was used to calculate a *p*-value determining the probability that the association between the genes in the data set and the canonical pathway was explained by chance alone. A pathway was considered significant with a *p*-value that was less than 0.05. All pathways were named according to the terminology used in IPA.

### Gene Set enrichment analysis of microarray data

GSEA was performed using gene sets from MSigDB v2.5 gene set database (http://www.broadinstitute.org/gsea/msigdb/). This analytical technique is designed to test a priori defined gene sets (for example, pathways) for association with phenotypes [32]. In brief, the method consists of the following steps: list of genes are first ranked by Signal2Noise metric using the correlation between their expression and the class distinction (for example, “before” versus “after” treatment samples). Given a defined set of genes (for example, genes members of a signalling pathway), the goal of GSEA is to determine whether the members of the gene set are found at the top or bottom of the list, indicating that they are associate with the phenotypic distinction, rather than being distributed uniformly or randomly across the list. An enrichment score is then calculated to quantify the degree to which a gene set is over-represented at the top or bottom of the entire ranked list. After calculation of the enrichment scores, statistical significance test was done with 1, 000 permutation of genes using the weighted to enrichment statistic.

### Quantitative real-time (qRT-PCR)

cDNA was generated from 500ng of total RNA using High Capacity RNA-to-cDNA kit (Life Technologies) according to the manufacturer’s instruction. qRT-PCR assays were performed using QuantiTect SYBR Green PCR kit and the following commercially available QuantiTect primers: (Qiagen, Crawley, UK) for *COX6C* (Assay ID QT00221137), *NDUFB2* (Assay ID QT00050904), and *LAT* (Assay ID QT00232127). Differences in expression were determined by the relative quantification method; the cycle threshold (C_T_) values of the target genes were first normalised to the C_T_ values of endogenous control large ribosomal protein P0 (*RPLPO*, Assay ID QT00075012).

## Results

### Patients

This exploratory analysis included only sJIA patients with active symptomatic disease who were selected on the basis that they responded (by attaining ACR90) to tocilizumab (Table 1). All the patients described herein additionally had normal CRP/ESR at second sample collection with an ACR90 response, and therefore all were considered as responders for the purposes of this study.

### Identification of genes/pathways associated with good response to tocilizumab

**Fig. 1:**
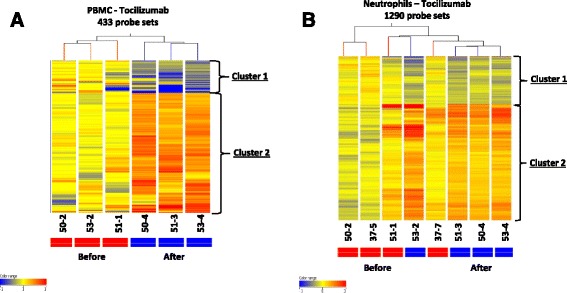
Heatmaps illustrating supervised hierarchical clustering analysis of the probe sets differentially expressed in PBMC (**a**) and Neutrophils (**b**) from sJIA patients before and after tocilizumab treatment (3 sJIA patients for PBMC samples and 4 sJIA patients for neutrophils). The differential expression of any probe set for a given gene was used as a surrogate for differential gene expression. Samples were collected at time points zero, and 3 months post treatment. All were responders with ACR90 (Table 1). Clustering analysis was performed in GeneSpring (GX11) as described in Methods. Normalized expression is colour coded in which red is high and blue is low relative to the median of the ‘before’ treatment samples. In the heatmaps, each column represents a sample and each row represents a gene. Full list of all genes in clusters are available in Additional files 1 and 2. However, listed below are genes with fold change (FC) ≥3 found within the clusters in the order of decreasing FC values. A cluster 1: Genes with significant decrease (FC ≥ 3 fold) after treatment with tocilizumab in PBMC: *FCGR3B, KCNJ15, CHI3L1, ADM, PROS1, SOCS3, CHI3L1, and NRG1*. A cluster 2: there were genes with increased expression on the heatmap but these were all less than 3 fold change. B cluster 1: Genes with significant decrease (FC ≥ 3 fold) after treatment with tocilizumab in neutrophils: *ARHGAP24, CLEC5A, TAF8.* B cluster 2: Genes with significant increase (FC ≥ 3 fold) after treatment with tocilizumab in neutrophils: *CD3D, LOC129293, AQP3, LAT, LY9, HLA-DPB1, TRA@, GFI1B, BCL11B, PASK, POLR3E, DOK2, AFG3L2, MEX3C, PASK, ENTPD6, KIAA0114, FAM102A, RCAN3, ATXN10, TNFAIP8L1, ABHD14B, RPL10A, GPR44, ATP6V0E2, ADARB1, APEX1, C17orf61, KLHL3, MRPS24, POU6F1, LDLRAP1, NDUFB2, SLC25A38, UBQLN4, KLF10, C22orf32, AKR1B1, PPP3CC, GSS, CAMK1, EIF3C, EEF2K, ILF3, RPL13, SLC25A6, THEM4, RPL13A, RDH14, KCTD15, DNMT1, TTC4, KIAA0748, AKR7A2, PLSCR3, ZNF639, KIAA1024, UNC84A, IARS, C11orf31, PVT1, DNPEP, LOC202781, LAGE3, NHP2, LSG1, SIRPG, SLC35B2, EEF2K, AES, TMEM14A, PAN2, DDX39, NOC4L, CAMSAP1, LOC100131731, BHLHE40, ECHS1, CLNS1A, CPSF1, LOC93622, TOMM5, COX6C, NLRC3, EIF3B, CIRH1A, OLIG1, ZBTB40*

After filtering out unreliable probe sets with expression at background level, 23,895 and 20,408 out of 54,459 probe sets were considered as expressed in PBMC and neutrophils respectively. Unsupervised hierarchical clustering (HC) of all expressed genes showed that the pre- and post-treatment samples from the patients (3 sJIA patients for PBMC samples and 4 sJIA patients for neutrophils) clustered together for each cell type (data not shown). Therefore, paired t-test of gene expression in each individual before and after treatment is preferred for the analysis of change over time (with statistical cut-off level of *p* < 0.05 with ≥ FC 1.5) to cross sectional analysis of the time points. The full lists of the genes are given in Additional files 1 (for PBMC) and 2 (for neutrophils). We performed supervised HC of the differentially expressed probe sets in PBMC and neutrophils separately, and generated heatmaps to illustrate the expression pattern between the two time points (Fig. 1a-b). This analysis yielded two main clusters of probe sets for each of the 2 cell types; cluster 1 for probe sets with decreased expression at 12 weeks, and cluster 2 corresponding to probe sets with increased expression at 12 weeks.

We used Ingenuity Pathway Analysis (IPA) tools to identify the significant pathways (*p* < 0.05) associated with the 2 lists of differentially expressed probe sets in PBMC and neutrophils. A summary of the results are presented in Table 2 for the top 10 IPA canonical pathways presented in order of significance.

**Table 2:** Top ten IPA pathways that were found to be significantly altered in PBMC and neutrophil samples from sJIA patients responding to tocilizumab

PBMC^a^	Neutrophils
Oncostatin M signalling (3)	Mitochondrial dysfunction (20)
Natural killer cell signalling (5)	EIF2 signalling (23)
Glutamine biosynthesis I (1)	NRF2-mediated oxidative stress response (20)
B Cell receptor signalling (6)	Calcium-induced T lymphocyte apoptosis (9)
PPARα/RXRα activation (6)	mTOR signalling (20)
Thyroid hormone biosynthesis (1)	Sucrose degradation V (Mammalian) (3)
	Regulation of eIF4 and p70S6K signalling (16)
	TCA cycle II (Eukaryotic) (5)
	CTLA4 signalling in cytotoxic T lymphocytes (11)
	Protein ubiquitination pathway (24)

^a^There were only 6 significant canonical pathways for this condition. In brackets are the numbers of genes from the input file in each pathway

#### PBMC gene expression profiling in tocilizumab treated patients showed changes in pathways associated with IL-6 signalling

Only 18 genes out of 385 differentially expressed unique genes in PBMC of sJIA patients were significantly mapped to 6 IPA canonical pathways (Table 2, Additional file 3). The most significant pathway was the Oncostatin M signalling pathway from this analysis; *p* = 0.015, with 3 mapped genes (*IL6ST*, *KRAS*, *CHI3L1*).

#### Neutrophil gene expression profiling demonstrated changes in mitochondrial and T cell pathways

The most significant change in IPA pathway for neutrophils was the “mitochondrial dysfunction pathway” (*p* = 0.000046) with increased expression of 20 genes in response to tocilizumab treatment (Table 2, Additional file 4). These genes included *ATP5A1*, an ATP synthase of complex V, and 13 other genes that code for subunits of other mitochondria respiratory chain complexes. In complex I there was increased expression of 8 nuclear encoded NADH dehydrogenase genes of which *NDUFB2* had the highest FC value of 3. Ubiquinol-cytochrome c reductase (*UQCRH*) coding for a hinge protein was the only gene regulated in complex III; in complex IV there was increased expression of nuclear encoded cytochrome c oxidase; *Cox5A*, *Cox6C* and *Cox7C*. The expression of linker for activated cells (*LAT*) was significantly increased 6-fold in response to tocilizumab treatment, and this gene was also mapped to two significant T cell pathways; CD28 Signalling in T Helper Cells and Regulation of IL-2 Expression in Activated and Anergic T Lymphocytes.

#### GSEA shows enrichment of mitochondrial/oxidative phosphorylation genes in sJIA patients treated with tocilizumab

**Fig. 2:**
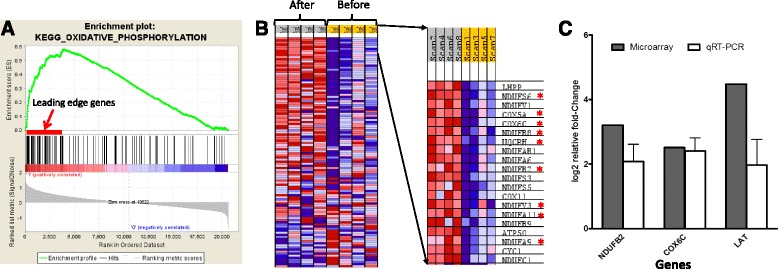
Genes associated with oxidative phosphorylation (mitochondria function). **a** Enrichment plot of KEGG_Oxidative phosphorylation gene set identified by GSEA. Middle section (black bars) illustrate the position of the genes belonging to the gene set in the context of all the genes on the Affymetrix U133 plus 2.0 array. The enrichment score (ES) plotted as a function of the position within the ranked list of array genes is shown as a green line. The ranked list metric shown in gray illustrates the correlation between the signal to noise values of all individually ranked genes according to the neutrophil samples of the ‘before’ and ‘after’ tocilizumab treatment (experimental conditions). **b** on the left is GSEA-derived heat map of the 110 leading edge genes that correlates with response to tocilizumab and contributing to the enrichment score; on the right is the top 20 genes that includes some of the genes mapped by IPA to mitochondria dysfunction pathway (in red asterisks). Signal intensities are illustrated by varying shades of red (increased) and blue (decreased). **c** Quantitative real-time polymerase chain reaction (qRT-PCR) validation of differentially expressed genes (*NDUFB2*, *COX6C*, *LAT*) observed in this microarray experiment. The relative fold change of both microarray (solid bar) and qRT-PCR (open bars) are shown. The data for qRT-PCR are the average of 3 independent experiments done on the same sample

We analysed the microarray data by GSEA [32] to determine whether the differential expression of mitochondrial genes observed in neutrophils of sJIA patients treated with tocilizumab were truly representative of the global gene expression data without regard to the accuracy of statistical threshold applied in the initial analysis. For GSEA, we extracted from MSigDB v2.5 gene set database (http://www.broadinstitute.org/gsea/msigdb/) the KEGGS_oxidative phosphorylation gene set which originally contained 135 genes, but the analysis was restricted to 110 genes present on the Affymetrix U133 plus 2.0 chip. This gene set was chosen because it is the best representative of the IPA mitochondrial dysfunction pathway as it contains 12 of the 20 genes mapped to this pathway (indicated with an asterisk in the Additional file 4). The result of GSEA showed significant enrichment of the oxidative phosphorylation gene set (Additional file 5 for the gene list) with increased expression of the leading edge gene subset in sJIA patients post tocilizumab treatment (Fig. 2a, b).

### Verification of Microarray analysis by qRT-PCR

Given the limitation in the blood sample volume, it was only possible to verify the accuracy of our microarray analysis by real-time quantitative PCR on just the neutrophil samples used in the array experiment. The qRT-PCR results confirmed the microarray data for *COX6C*, *LAT* and *NDUFB2* (Fig. 2c).

### Minor change in neutrophil activation following sample manipulation

Before the microarray analysis of gene expression in neutrophils, we carried out a series of control experiments where we compared expression level of the neutrophil activation marker CD11b/Mac-1 measured in geometric mean fluorescence intensity (GMFI) on CD16+ cells gated on granulocytes population within whole blood to that of isolated neutrophils ±1μg/ml LPS by flow cytometry. These experiments were performed in healthy controls as well as sJIA patients with active and inactive disease. There was a small increase in CD11b expression due to the separation process. However, neutrophils were still relatively inactive since they were highly sensitive (>10 fold increase) to further activation when stimulated with LPS in both healthy controls and sJIA patients (Additional file 6). The purity of isolated neutrophils as defined from the double positive cells was >96 % (Additional file 7). The median ± standard deviation (SD) of double positive isolated neutrophils for all the sJIA samples was 96.55 % ± 0.63 compared to 60 % ± 6.89 in whole blood (Additional file 7).

## Discussion

A novel and important aspect of this study is that we examined neutrophils in addition to PBMC since these cells are known to play important roles in innate immune responses and the pathogenesis of sJIA [33–35], but previously have not been studied by transcriptome analyses in this context. Both IPA and GSEA showed significant correlation between mitochondrial/oxidative stress genes and response to tocilizumab in neutrophils of sJIA patients. Our microarray data indicated a significant increase in the expression levels of nuclear encoded mitochondrial genes that are involved in electron transport (e.g. *NDUFB2*, *COX6C,* both confirmed by q RT-PCR) after a good clinical response to tocilizumab; or conversely, that decreased gene expression was present in those with active disease. This observation is consistent with the study by Ishikawa et al. using whole blood for gene profiling, showing reduced expression of mitochondrial DNA-encoded genes in sJIA patients with active disease compared to healthy controls [18]. They did not detect differences in any of the nuclear encoded genes including those found in this study, which might be due to differences in study design, analysis of a mixed cell population of whole blood (PAXgene) samples, microarray platforms and/or analytical methods used. Our data are further supported by the fact that IL-6 has been shown to have a direct effect on mitochondrial function by decreasing both the membrane potential and ATP production with subsequent increase in intracellular reactive oxygen species (ROS) level in an in vitro study [36]. Currently, there is growing evidence that mitochondrial ROS and defective antioxidant responses play an important role in the pathogenesis of a number of autoinflammatory and autoimmune diseases [37–42]. Given that IL-6 is one of the most important mediators of fever and the acute phase response (including neutrophilia), and that neutrophil mitochondrial ROS are increasingly understood to be important in autoinflammation, it is perhaps unsurprising that we have detected perturbation of mitochondrial genes in neutrophils in active sJIA, which change in response to successful therapy with IL-6 blockade. This further emphasises the potential importance of studying neutrophils in sJIA and its response to treatment in the future.

It was striking and unexpected to observe the differential expression of T-cell receptor associated genes such as delta 3 molecule (*CD3D*), zeta-chain tyrosine kinase (*Zap70*) and linker for activation of T cell (*LAT*, confirmed by q RT-PCR) in the neutrophil cell population from patients treated with tocilizumab. Although we cannot rule out minor and uneven contamination of the separated cell populations by T cells, previous studies have revealed that human neutrophils do express TCR including components of the signalling complex [43, 44], but their exact role in neutrophils is not yet known. Our microarray data showed that *LAT* is expressed in neutrophils of all the 4 patients studied. Furthermore Puellmann et al. addressed the issue of contamination by clearly demonstrating that the neutrophil cell populations were negative for T cell markers (CD3, CD4, and CD8) [44]. These findings suggest that neutrophils may have the potential to mediate both innate and adaptive immune responses in sJIA patients.

It was reassuring to note that in the DEGs from the PBMC of tocilizumab-treated sJIA patients, there was decreased expression of *SOCS3*, a suppressor of cytokine signalling, consistent with the fact that tocilizumab decreases intracellular IL-6 signalling and therefore reduction in the expression of *SOCS3*. We have previously reported that specific innate immune genes are upregulated in PBMC of sJIA patients during active disease [2]. In the same study we also demonstrated that B cells express higher levels of IL-6 than monocytes. Given the heterogeneous composition of PBMC samples, it is possible that the significant B cell pathway and oncostatin M signalling observed in this study might be from B cells, further illustrating the importance of examining a homogeneous population of cells.

One obvious limitation of this study is that the sample sizes are small, and therefore deemed likely to generate a number of false positive genes. This effect is alleviated to some extent by a prospective approach in sample collection and paired sample analyses. The independent application of GSEA provided a strong supporting evidence for the observed increased expression of mitochondrial gene sets following good response to tocilizumab, thereby validating this finding. We have also identified genes/pathways previously known to be involved in the pathogenesis of sJIA, suggesting that our findings are not false positives.

It might be potentially important and interesting to profile patients who do not clinically respond to IL-6R blockade, assuming that their pathology is via a different pathway. We have not addressed this question in this initial study because the lack of the clinical response to tocilizumab is a continuum that may be due to many reasons. We feel that using a clinically well-defined full response to drug therapy of ACR90 is better to observe significant differences and also allows us to collect sufficiently similar patients to do statistical comparisons.

## Conclusions

In conclusion, we have shown that mitochondrial genes in neutrophils could play a role in the pathogenesis of some sJIA patients, and highlight that it is important to examine the neutrophils in future transcriptome analyses in sJIA, as has already been shown in polyarticular JIA [21-23, 45, 46].

## Additional files

Additional file 1:
**File contains the list of 433 significant probe sets obtained by paired t-test using the statistical cut-off level of** ***p*** **< 0.05 with FC ≥ 1.5 in PBMC from sJIA patients successfully treated with tocilizumab.** (XLS 86 kb) Additional file 2:
**File contains the list of 1290 significant probe sets obtained by paired t-test using the statistical cut-off level of** ***p*** **< 0.05 with FC ≥ 1.5 in neutrophils from sJIA patients successfully treated with tocilizumab.** (XLS 227 kb) Additional file 3:
**File contains the significant IPA canonical pathways obtained in PBMC from sJIA patients successfully treated with tocilizumab.** (XLSX 12 kb) Additional file 4:
**File contains the significant IPA canonical pathways obtained in neutrophils from sJIA patients successfully treated with tocilizumab.** (XLSX 22 kb) Additional file 5:
**File contains the enriched genes for KEGG_Oxidative Phosphorylation pathway obtained from GSEA.** (XLS 13 kb) Additional file 6:
**Changes in Neutrophil CD11b/Mac-1expression following sample manipulation and LPS stimulation.** We compared expression level of CD11b/Mac-1 on the surface of CD16+ cells gated on the granulocyte population within whole blood to those of isolated neutrophils ±1μg/ml LPS. We examined 4 individual controls, 3 systemic patients with active disease (denoted ‘A’) and two with inactive disease (denoted ‘I’). (PPTX 65 kb) Additional file 7:
**Flow cytometry analysis of neutrophils from sJIA patients.** A: representative dot plots of forward and side scatter of unstained whole blood and isolated neutrophils gating on granulocytes. B: evaluation of human cells stained with antihuman CD16-Cy7 (y-axis) and CD11b/Mac-1-PE (x-axis) conjugated antibodies in whole blood (left panel) and isolated neutrophils (right panel). The percentages of positive cells are indicated in each quadrant. The percentage of double positive cells (B; top right quadrant) is higher in isolated neutrophils (96.5 %) than in whole blood (58.9 %). C: FACS analysis data for isolated neutrophils and whole blood (WB) samples obtained from 4 sJIA patients before and after tocilizumab treatment. Values on the y-axis corresponds to percentages of double positive CD11bMac-1+ CD16+ cells. The horizontal bars show median values. (PPTX 49 kb)

## Abbreviations

ACR: American College of Rheumatology
DEGs: differentially expressed genes
ILAR: International League of Associations for Rheumatology
MTX: methotrexate
PBMC: peripheral blood mononuclear cells
sJIA: Systemic Juvenile Idiopathic Arthritis
