# Supplementary figures and images for: Mitochondrial and oxidative stress genes are differentially expressed in neutrophils of sJIA patients treated with tocilizumab: a pilot microarray study
Source: Pediatr Rheumatol Online J. 2016 Feb 9;14:7. doi: 10.1186/s12969-016-0067-7 (PMC4746827; doi:10.1186/s12969-016-0067-7)

## Slide 1
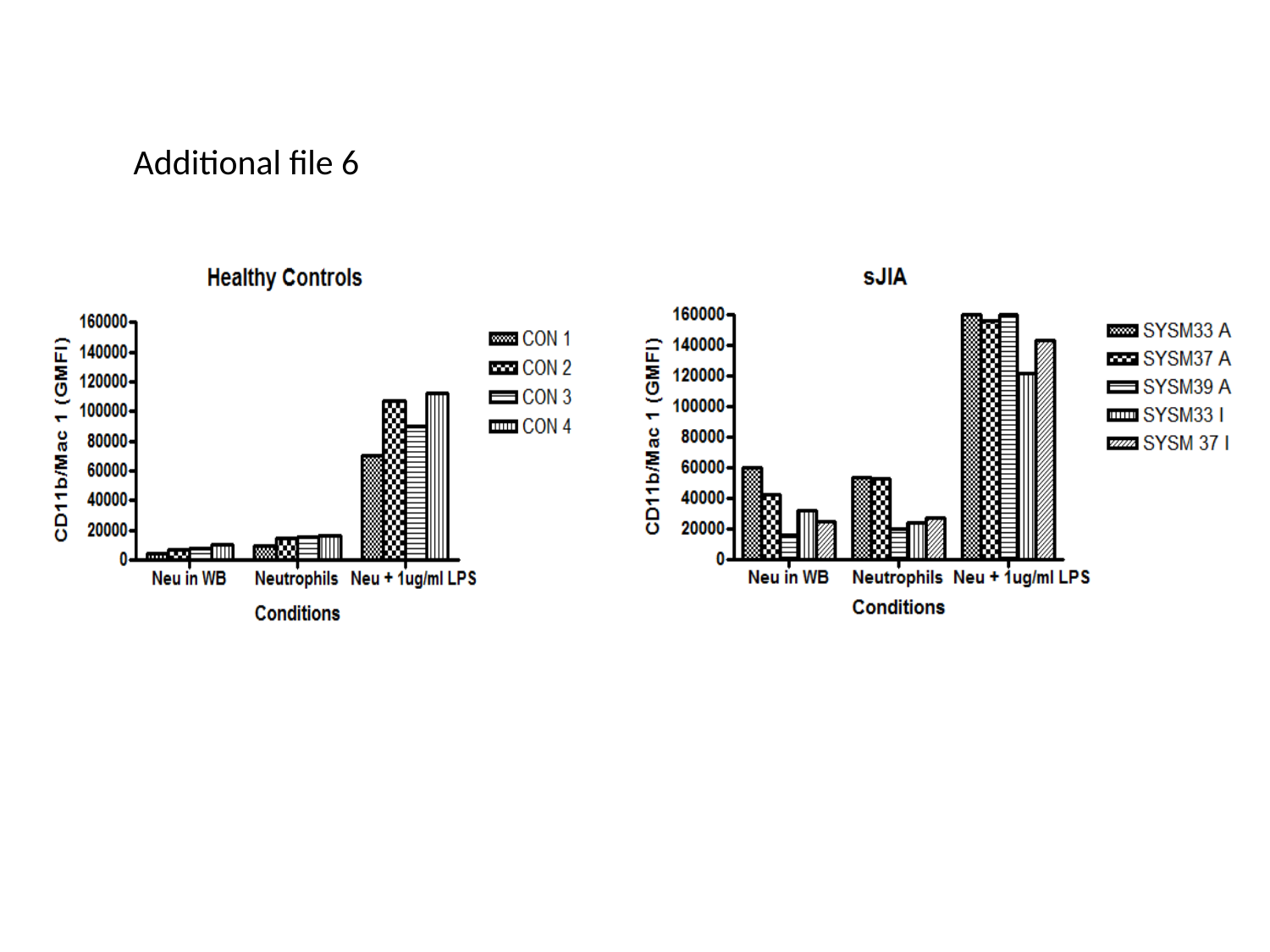

Additional file 6

Supplement: Additional file 6: — Changes in Neutrophil CD11b/Mac-1expression following sample manipulation and LPS stimulation. We compared expression level of CD11b/Mac-1 on the surface of CD16+ cells gated on the granulocyte population within whole blood to those of isolated neutrophils ±1μg/ml LPS. We examined 4 individual controls, 3 systemic patients with active disease (denoted ‘A’) and two with inactive disease (denoted ‘I’). (PPTX 65 kb) [file 12969_2016_67_MOESM6_ESM.pptx]

## Slide 1
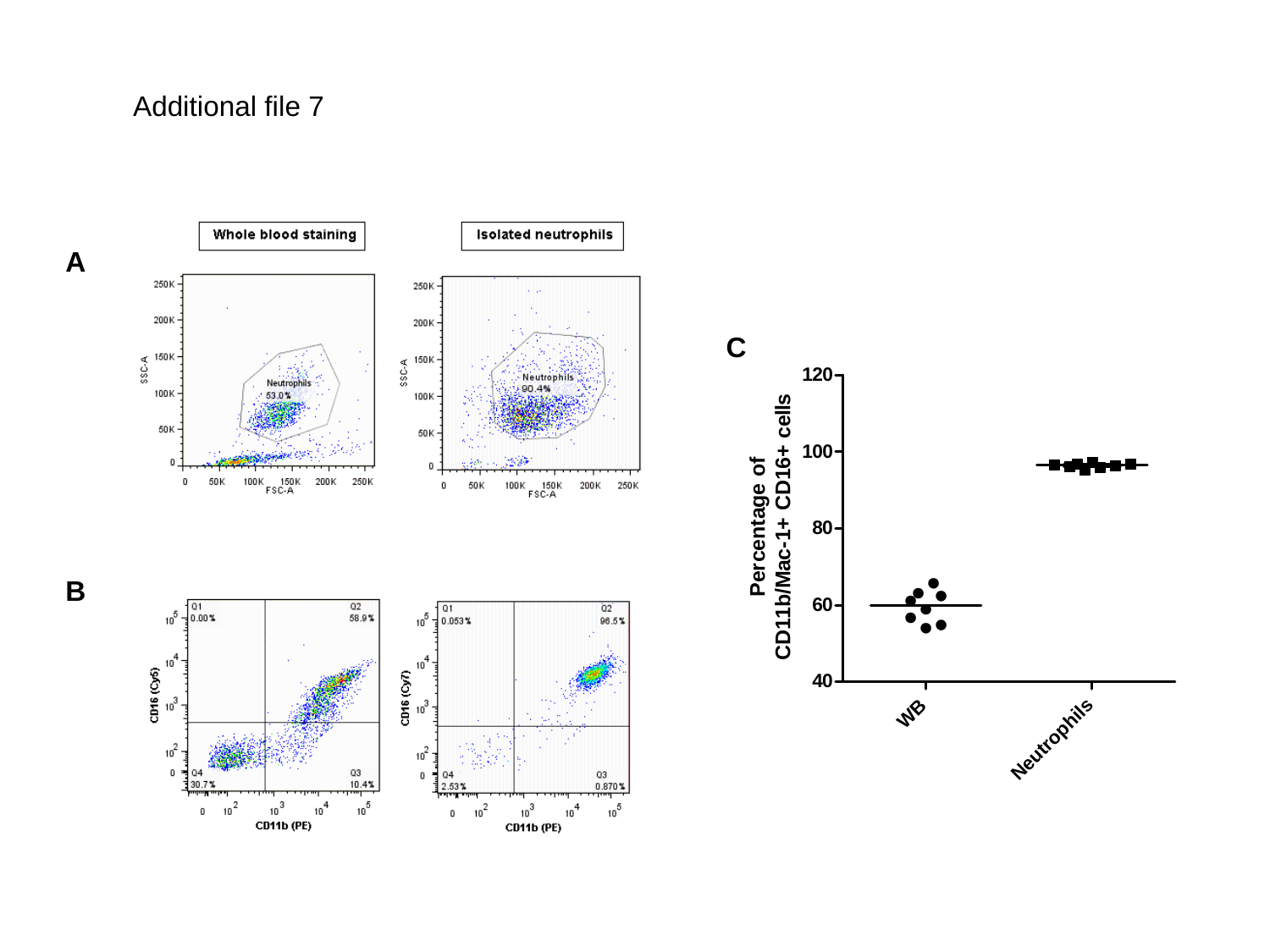

Additional file 7
A
C
B

Supplement: Additional file 7: — Flow cytometry analysis of neutrophils from sJIA patients. A: representative dot plots of forward and side scatter of unstained whole blood and isolated neutrophils gating on granulocytes. B: evaluation of human cells stained with antihuman CD16-Cy7 (y-axis) and CD11b/Mac-1-PE (x-axis) conjugated antibodies in whole blood (left panel) and isolated neutrophils (right panel). The percentages of positive cells are indicated in each quadrant. The percentage of double positive cells (B; top right quadrant) is higher in isolated neutrophils (96.5 %) than in whole blood (58.9 %). C: FACS analysis data for isolated neutrophils and whole blood (WB) samples obtained from 4 sJIA patients before and after tocilizumab treatment. Values on the y-axis corresponds to percentages of double positive CD11bMac-1+ CD16+ cells. The horizontal bars show median values. (PPTX 49 kb) [file 12969_2016_67_MOESM7_ESM.pptx]
